# Comparison of two malaria multiplex immunoassays that enable quantification of malaria antigens

**DOI:** 10.1186/s12936-022-04203-9

**Published:** 2022-06-07

**Authors:** Ihn Kyung Jang, Alfons Jiménez, Andrew Rashid, Rebecca Barney, Allison Golden, Xavier C. Ding, Gonzalo J. Domingo, Alfredo Mayor

**Affiliations:** 1 Diagnostic Group, PATH, 2201 Westlake Ave, Suite 200, Seattle, WA 98121 USA; 2 ISGlobal, Barcelona Institute for Global Health, Hospital Clínic—Universitat de Barcelona, Carrer Rosselló 153 (CEK Building), 08036 Barcelona, Spain; 3 Spanish Consortium for Research in Epidemiology and Public Health (CIBERESP), Av. Monforte de Lemos, 3-5, Pabellón 11, 28029 Madrid, Spain; 4 The Foundation for Innovative New Diagnostics, Campus Biotech, 9 Chemin des Mines, 1202 Geneva, Switzerland; 5 Centro de Investigação Em Saúde de Manhiça Rua 12, Cambeve CP, 1929 Maputo, Mozambique; 6 Department of Physiologic Sciences, Faculty of Medicine, Universidade Eduardo Mondlane, Maputo, Mozambique

**Keywords:** Malaria, Immunoassay, Multiplex, HRP2, pLDH, *P. falciparum*, *P. vivax*, *P. malariae*, *P. knowlesi*, *hrp2/hrp3* deletions, Standardization

## Abstract

**Background:**

Immunoassay platforms that simultaneously detect malaria antigens including histidine-rich protein 2 (HRP2)/HRP3 and *Plasmodium* lactate dehydrogenase (pLDH), are useful epidemiological tools for rapid diagnostic test evaluation. This study presents the comparative evaluation of two multiplex platforms in identifying *Plasmodium falciparum* with presence or absence of HRP2/HRP3 expression as being indicative of *hrp2/hrp3* deletions and other *Plasmodium* species. Moreover, correlation between the malaria antigen measurements performed at these platforms is assessed after calibrating with either assay standards or international standards and the cross-reactivity among *Plasmodium* species is examined.

**Methods:**

A 77-member panel of specimens composed of the World Health Organization (WHO) international *Plasmodium* antigen standards, cultured parasites for *P. falciparum* and *Plasmodium knowlesi*, and clinical specimens with mono-infections for *P. falciparum*, *Plasmodium vivax*, and *Plasmodium malariae* was generated as both whole blood and dried blood spot (DBS) specimens. Assays for HRP2, *P. falciparum*–specific pLDH (*Pf*LDH), *P. vivax*–specific pLDH (*Pv*LDH), and all human *Plasmodium* species Pan malaria pLDH (PanLDH) on the Human Malaria Array Q-Plex and the xMAP platforms were evaluated with these panels.

**Results:**

The xMAP showed a higher percent positive agreement for identification of *hrp2*-deleted *P. falciparum* and *Plasmodium* species in whole blood and DBS than the Q-Plex. For whole blood samples, there was a highly positive correlation between the two platforms for *Pf*LDH (Pearson *r* = 0.9926) and *Pv*LDH (*r* = 0. 9792), moderate positive correlation for HRP2 (*r* = 0.7432), and poor correlation for PanLDH (*r* = 0.6139). In Pearson correlation analysis between the two platforms on the DBS, the same assays were *r* = 0.9828, *r* = 0.7679, *r* = 0.6432, and *r* = 0.8957, respectively. The xMAP HRP2 assay appeared to cross-react with HRP3, while the Q-Plex did not. The Q-Plex *Pf*LDH assay cross-reacted with *P. malariae*, while the xMAP did not. For both platforms, *P. knowlesi* was detected on the *Pv*LDH assay. The WHO international standards allowed normalization across both platforms on their HRP2, *Pf*LDH, and *Pv*LDH assays in whole blood and DBS.

**Conclusions:**

Q-Plex and xMAP show good agreement for identification of *P. falciparum* mutants with *hrp2/hrp3* deletions, and other *Plasmodium* species. Quantitative results from both platforms, normalized into international units for HRP2, *Pf*LDH, and *Pv*LDH, showed good agreement and should allow comparison and analysis of results generated by either platform.

**Supplementary Information:**

The online version contains supplementary material available at 10.1186/s12936-022-04203-9.

## Background

The standard of care for malaria diagnosis is blood smear microscopy and antigen detection through rapid diagnostic test (RDT). Microscopy has limitations in terms of difficulty in identifying mixed infections, and in user expertise and training requirements [1]. RDTs are more amenable for the diagnosis of malaria in settings with limited laboratory infrastructure where the majority of the malaria disease burden lies.

Several biomarkers, including *Plasmodium falciparum*–specific histidine-rich protein 2 (HRP2), *Plasmodium* lactate dehydrogenase (pLDH), and *Plasmodium* aldolase (pAldo), have been demonstrated to provide the discriminatory ability for detecting malaria parasites and classifying *Plasmodium* species [2]. In particular, the presence of a pan-epitope and species-specific epitopes on pLDH provides a tool to detect *Plasmodium* parasite as well as to classify the specific parasite species, such as *P. falciparum* and *Plasmodium vivax*, which are the major causes of human malaria. HRP2 has a high turnover rate during the asexual cycle and has an extended half-life in blood compared to pLDH, resulting in it being more abundant during malaria infection [3–5]. As such, the most widely used RDTs for *P. falciparum* target HRP2 and a homologous protein HRP3. HRP2 and HRP3 are nonessential proteins, and *P. falciparum* mutants with deletion of either or both genes coding for these proteins have been increasing in prevalence in malaria endemic countries, impacting the sensitivity and utility of HRP2-based tests in these settings [6].

The gold standard for malaria detection is confirmation of the presence of parasite DNA or RNA in whole blood by Polymerase chain reaction (PCR) testing. RDTs detect parasite antigens in whole blood, the presence of which does not fully correlate with that of the parasite nucleic acid. Quantitative enzyme-linked immunosorbent assays (ELISA), targeting the diagnostic malaria antigens, provide an independent approach for confirming the presence of malaria antigens in samples and therefore can inform the evaluation of RDTs [7, 8]. ELISA technologies that are capable of multiplexing offer many key qualities, including high-throughput potential, more results per sample, and lower sample volumes. Several laboratories have developed the tools to simultaneously quantify malaria antigens using two different technology platforms: the planar-based array and the magnetic bead-based platforms. Today there is only one commercial multiplexed assay for malaria antigen quantification, which is a planar array-based platform (the Q-Plex technology) and detects five biomarkers: HRP2, pan-specific pLDH (PanLDH), *P. falciparum*–specific pLDH (*Pf*LDH), *P. vivax*–specific pLDH (*Pv*LDH), and C-reactive protein (CRP) in whole blood and DBS [9, 10]. The bead-based platform using the xMAP technology has been applied for the development of two noncommercial multiplex malaria antigen assays targeting HRP2, PanLDH, *Pf*LDH, *Pv*LDH and pAldo in whole blood, plasma, and dried blood spot (DBS) [11–13].

In addition to identifying *P. falciparum* mutants with *hrp2* deletion, these two multiplexed assays have been used for the investigation of the dynamics of antigen clearance, conducting malaria surveillance studies, anti-malarial drug clinical trials, and evaluating point-of-care tests [8, 14–16]. The different multiplexed assays use different antibody reagents as well as different calibration standards, resulting in a difference in overall performance and quantification across platforms. There is a recognition in the malaria community for the need to align assay results with international antigen standards. Recently, the National Institute for Biological Standards and Control (NIBSC) established lyophilized World Health Organization (WHO) international standards for *P. falciparum* and *P. vivax* antigens in order to ensure accurate results and the quality of malaria tests [17, 18]. This study sought to compare the performance of two multiplex assays for detecting malaria antigens in serially diluted samples (whole blood or DBS) that were prepared by spiking WHO international standards, parasite culture, or clinical samples with a view to verifying the compatibility of data between both platforms. The assays were the Human Malaria Array hosted on the Q-Plex platform (Quansys, Logan, UT, USA) and the multiplex bead-based assay on the xMAP platform developed by ISGlobal.

## Methods

### Reagents

The WHO international standards for *P. falciparum* (product code: 16/376) and *P. vivax* (product code: 19/116) antigens were purchased from the NIBSC (Hertfordshire, UK). *Plasmodium falciparum* W2 (*hrp2*^+^*hrp3*^+^) and Dd2 (*hrp2*^*–*^*hrp3*^+^) strains were obtained from Biodefense and Emerging Infections Research Resources Repository (BEI) Resources (Manassas, VA, USA) and the 3BD5 (*hrp2*^*–*^*hrp3*^*–*^) strain was obtained from the National Institute of Allergy and Infectious Diseases (Bethesda, Maryland, USA). *Plasmodium knowlesi* strain A1-H2 was a kind gift from Dr. Rob Moon (London School of Hygiene and Tropical Medicine, UK). Clinical specimens for *P. falciparum, P. vivax,* and *Plasmodium malariae* were acquired from Discovery Life Sciences (Santa Barbara, CA, USA). For calibration standards of the xMAP platform, HRP2 protein was purchased from Microcoat Biotechnologies’ (Starnberger See, Germany), and recombinant *Pf*LDH and *Pv*LDH proteins were purchased from MyBioSource (San Diego, CA, USA), Relia-Tech (Santa Fe Springs, CA, USA), and Microcoat. Pooled ethylenediaminetetraacetic acid–anticoagulated blood from five O + donors was used to prepare the sample panels.

### Culture

*Plasmodium falciparum* and *P. knowlesi* laboratory strains were in vitro cultured according to procedures described previously [19, 20]. Synchronization of *P. falciparum* and *P. knowlesi* cultures was performed by D-sorbitol treatment or gradient centrifugation procedures using Nycodenz^®^ solution (Axis-shield Diagnostics Ltd, Scotland) in 10 mM HEPES (pH 7.0), respectively [20, 21]. Parasitaemia was determined via staining of smear and light microscopy with a 100 × oil objective.

### Sample panel preparation

The two-fold dilution series of 67 samples were prepared after spiking the material from the aforementioned antigen sources in pooled blood and aliquoting into cryovials, which were then stored at − 80 °C in a PATH laboratory until use. DBS samples were subsequently prepared from matched blood samples by spotting 60 µL of blood onto Whatman^®^ 903 protein saver cards (GE Healthcare, Chicago, IL, USA), drying at room temperature overnight, and then storing at − 20 °C in sealed plastic pouches containing desiccant packets as described previously [10]. The sample panels consisting of 67 samples in frozen blood and DBS (Table 1), except those prepared with the WHO international standard *P. vivax* antigen, were sent to the ISGlobal laboratory, where the testing occurred. To limit the potential variability arising from differences in sample integrity that could happen during the transport, a set of the sample panels was subjected to a similar shipping time and storage conditions. Ten samples with WHO international standard *P. vivax* antigen were independently prepared in whole blood and DBS by each laboratory (Table 1).

**Table 1:** List of spiked samples in the blood and DBS panels used in this study

Category	Samples	Source	Type	Dilution	Matrix	# of samples
International standard	WHO international standard *P. falciparum* antigen	NIBSC	Cell culture	9-points + 1 blank	WB, DBS	10
	WHO international standard *P. vivax* antigen	NIBSC	Pool of clinical	9-points + 1 blank	WB, DBS	10
Detection of failed HRP2/HRP3 expression	*P. falciparum*, W2 (*hrp2* + *hrp3* +)	PATH	Cell culture	5-points	WB, DBS	5
	*P. falciparum*, Dd2 (*hrp2–hrp3* +)	PATH	Cell culture	5-points	WB, DBS	5
	*P. falciparum*, 3DB5 (*hrp2–hrp3–*)	PATH	Cell culture	5-points	WB, DBS	5
Reactivity to native proteins	*P. falciparum*	Discovery life science	Clinical	5-points	WB, DBS	15
	*P. vivax*	Discovery life science	Clinical	5-points	WB, DBS	10
	*P. malariae*	Discovery life science	Clinical	5-points	WB, DBS	10
	*P. knowlesi* (A1-H1)	PATH	Cell culture	5-points	WB, DBS	5
Baseline specificity	Negative (a pool of 5)		NA	NA	WB, DBS	2
Total						77

*WB* whole blood, *DBS* dried blood spot, *NA* not applicable

### Q-Plex platform procedure

The 5-Plex was run as per the manufacturer’s instructions. All whole blood and DBS samples were subjected to blinded experiments while performing the Q-Plex platform procedure. Whole blood samples were directly used without any manipulation, while the eluates were used for DBS by first incubating a 6 mm disc punched out from each DBS in elution buffer overnight as described previously [10]. Calibrators and samples were prepared according to the manufacturer’s protocol. Each sample was tested both neat and diluted 20-fold in single wells unless noted otherwise. After the addition of 50 μL of calibrators and samples, the plate was incubated at room temperature with shaking at 500 revolutions per minute (rpm) for 2 h. Plates were then washed with a proprietary wash buffer using an automated plate washer. A 50 μL aliquot of detection mix, including biotinylated detector antibody and buffer, was added to each well, and the plate was incubated with shaking for another hour and then washed again. For detection, a 50 μL aliquot of horseradish peroxidase–streptavidin solution was added to each well and then incubated with shaking for 30 min. After a final wash, a 50 μL aliquot of chemiluminescent substrate solution was added to each well, and the chemiluminescent intensity from the array spots in each well was immediately measured using the Q-View Imager Pro (Quansys Biosciences, Logan, UT, USA) at an exposure time of 300 s. The antigen concentrations were calculated using a five-parameter logistic fit model, which was built into the Q-View Software (Quansys Biosciences).

### Luminex platform procedure

HRP2, *Pv*LDH, and *Pf*LDH were run in a 3-Plex Luminex assay format, and PanLDH was run separately in singleplex to avoid cross-reactivity with the species-specific pLDH assays, as described previously [13]. Briefly, following the same scheme of “Q-Plex platform procedure,” all blinded whole blood and DBS samples were assayed on the xMAP platform. Whole blood samples were directly used without any manipulation, while DBS samples were eluted by incubating a 3 mm disc punched out from each DBS in Luminex buffer ([LB] 1% bovine serum albumin in phosphate-buffered saline [PBS] with sodium azide at 0.05%), at 50 µL per punch, overnight at 4 °C in gentle agitation. Previously, sets of MagPlex^®^ Microspheres (Luminex Corp., Austin, TX, USA) with set-specific spectral signatures were coupled with the respective monoclonal capture antibodies: anti-*Pv*LDH (PA8, Access Bio, Somerset, New Jersey, USA), anti-*Pf*LDH (PA11, Access Bio), anti-PANpLDH (PA12, Access Bio), anti-HRP2 (MBS832975, MyBioSource), according to the manufacture’s protocol at 30 µg/mL of microspheres.

The assay was performed by incubating 50 μL of microsphere suspension in LB (1000 microspheres of each/well in the 3-Plex or 2000 microspheres/well in the singleplex) with 50 μL of each sample, which was tested both neat (twofold final dilution per well) and diluted 20-fold in LB (40-fold final dilution per well) unless noted otherwise, overnight at 4 ºC with shaking at 600 rpm in the dark. Plates were then washed with wash buffer (0.05% Tween 20 [Sigma-Aldrich, St. Louis, MO, USA] in PBS) after first pelleting microspheres using a magnetic separator (EMD Millipore, Burlington, MA, USA). A 100 μL aliquot of detection mix containing biotinylated antibodies (EZ-Link Sulfo-NHS-Biotin Kit, Thermo Fisher Scientific, Waltham, MA, USA) to anti-PANpLDH (PA12, Access Bio) and anti-HRP2 (MBS834434, MyBioSource) for the 3-Plex assay and anti-PANpLDH (PA2, Access Bio) for the singleplex assay, all of them biotinylated in the lab using the EZ-Link Sulfo-NHS-Biotin Kit (21,435, Thermo Fisher Scientific, Waltham, MA, USA) according to manufacturer’s instructions, were added to each well at 1:1000 dilution in LB, and the plate was incubated with shaking for 1 h in the dark and then washed again. For detection, 100 µL of Streptavidin-R-Phycoerythrin (42,250, Sigma-Aldrich) at 1:1000 dilution in LB was added to each well and then incubated with shaking for 30 min in the dark. Finally, the beads were washed and resuspended in LB, and the plate was read using the Luminex xMAP 100/200 analyzer (Luminex Corp.). Fifty microspheres for each antigen were read, and the result was given as median fluorescence intensity (MFI). The antigen concentrations were calculated using a five-parameter logistic fit model using GraphPad Prism (version 6, GraphPad Software, San Diego, CA, USA). The characteristics of the xMAP and Q-Plex assay are compared in Additional file 1: Table S1.

### Statistics

Whole blood and DBS eluate samples were analysed in singlet in both neat and 20-fold dilution by each assay. The working assay range was established as the range at which precision stayed under 20% of the coefficient of variation (CV) between adjusted measured concentration values of two adjacent levels. For the Q-Plex platform, the lower limit of detection (LLOD), upper limit of quantification (ULOQ), and lower limit of quantification (LLOQ) were previously described [9]. For the xMAP platform, LLOD and LLOQ were calculated using the formulas mean (blanks) + 3 × standard deviation (blanks) and mean (blanks) + 6 × standard deviation (blanks), respectively [13]. The assay characteristics including cutoff, LLOD, LLOQ, and ULOQ for HRP2, *Pf*LDH, *Pv*LDH, and PanLDH for the Q-Plex and xMAP platform are summarized in Additional file 1: Table S2. Since CRP assay is only incorporated into the Q-Plex platform, CRP data was not included for analysis. The dilutional linearity and inter-assay variability were determined using the WHO international standards for *P. falciparum* or *P. vivax* antigens. The dilutional linearity was assessed for the ability to generate results that have a linear response, proportional to the concentration of the international standard. The WHO international standards for *P. falciparum* and *P. vivax* antigens, ranging from 1.6 to 400 IU/mL, were used to evaluate the performance of the HRP2, *Pf*LDH, PanLDH, and *Pv*LDH assays with each assay-specific calibrator set. The linear range of the curve was estimated using the least squares method analyzing the regression coefficient (R^2^).

The inter-assay variability, a measure of the degree of reproducibility, was calculated using the CV value with a formula: (standard deviation/mean) × 100. This was determined by repeated analysis of pixel or fluorescence intensity values from identical samples of the WHO international standard *P. falciparum* and *P. vivax* antigens at 50 IU/mL, 25 IU/mL, 12.5 IU/mL, and 6.3 IU/mL concentration over multiple days for the Q-Plex and xMAP platforms, respectively. Samples were run three times over a week. The acceptable level of inter-assay variability was defined as ≤ 15% for whole blood and DBS. If necessary, the inter-assay variability was assessed using the extrapolated antigen concentration values under the same acceptance criteria (≤ 15%).

Diagnostic concordance in the identification of *P. falciparum* with *hrp2/hrp3* deletions and *Plasmodium* species was estimated using the established cutoffs (mass concentrations in pg/mL). For the Q-Plex, platform cutoffs yielding 99.5% or more diagnostic specificity were used [9]. For the xMAP platform, cutoffs were calculated using the mean of negative controls + 3 standard deviations. Positive agreement (percentage) was calculated by examining the proportion of reference positive results in which the test result is positive.

Pearson correlation coefficient was used to evaluate the relationship of antigen concentrations obtained by the two platforms. Samples with concentration of antigen < lower limit of detection were excluded from the analyses. Bland–Altman plots were used to compare the difference in agreement with antigen amounts (y-axis) with the average of antigen amounts from two assays (x-axis). To assess differences in malaria antigen levels measured by the two multiplex assays, *p-*values were calculated using the unpaired t-test. Statistical analyses were performed using GraphPad Prism software version 6 (GraphPad Software). Any *p*-value of ≤ 0.05 was considered statistically significant.

## Results

### Linearity and reproducibility

**Fig. 1:**
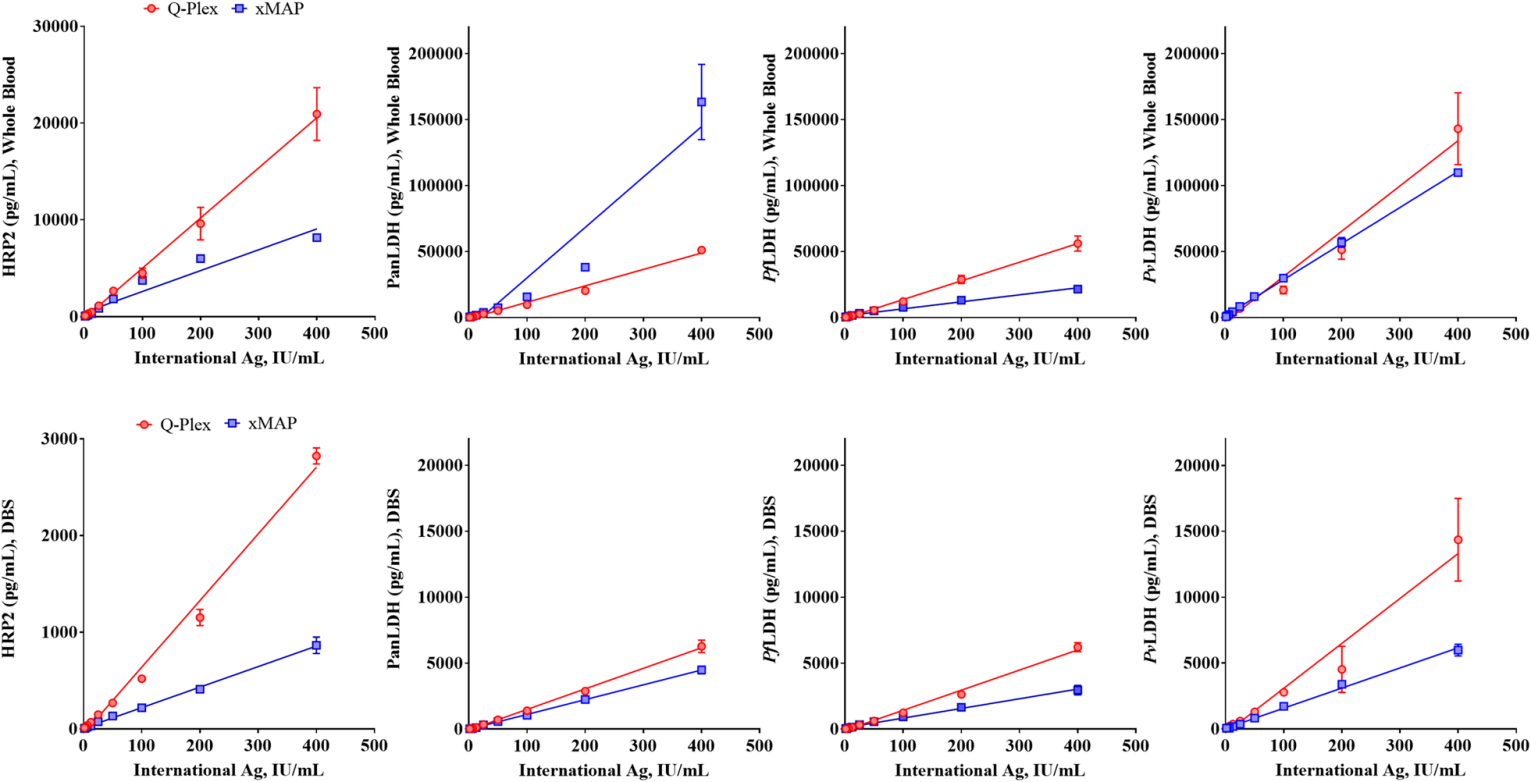
Dilutional linearity of antigens from the WHO international *P. falciparum* and *P. vivax* antigens. The WHO international *P. falciparum* and *P. vivax* antigen standards in the range of 1.6 to 400 IU/mL were evaluated by one operator in three independent experiments over a week and plotted as mean of analyte concentration ± standard deviation (SD) for (**A**) whole blood and (**B**) DBS. The y-axis of each plot represents the concentrations of target antigen calculated using the calibrators of the respective assay. Dilutional linearity was determined from regression analysis of observed mass concentrations versus the expected concentrations in international unit. Red symbol—Antigens estimated by using the WHO international standard *P. falciparum* antigen. Blue symbol—Antigens estimated by using the WHO international standard *P. vivax* antigen. The best-fit trend line for each individual antigen is shown in its color code

A two-fold dilution series of the respective international standard antigen in whole blood and DBS was tested to assess linearity across the range of 0.16 to 400 IU/mL for each assay and examine the relationship between assay results and the WHO international standard antigens. All four assays (HRP2, PanLDH, *Pf*LDH and *Pv*LDH were linear in the range of 1.6 to 400 IU/mL on both platforms (Fig. 1). The equations for the best-fit lines are summarized for all antigens in Additional file 1: Table S3. The best-fit slope values for the respective antigen assays show a wide difference between the two platforms because the antigen quantification can be largely influenced by values assigned to the standards, but the R^2^ values of the curves for each of the assays were in the range of 0.9461–0.9885 and 0.9215–0.9938 for whole blood and DBS-based Q-Plex data, respectively, and 0.9037–0.9980 and 0.9796–0.9947 for whole blood and DBS-based xMAP data (Additional file 1: Table S3). Both datasets from the Q-Plex and xMAP assays demonstrated a high degree of linearity as well as correlation for all antigens.

To determine the reproducibility of the data, which should not be influenced by day-to-day variation, inter-assay performance of both platforms was measured by quantifying the CV of the assay results using the international standard antigen samples prepared in whole blood and DBS, which were tested on three different days over a week (Table 2**)**. Overall, the minor variability in the results of the whole blood samples indicate that these two platforms were highly reproducible, with the average CV less than 10% across all platforms and all antigens. The inter-assay variability for HRP2 and *Pf*LDH measurement using DBS samples was within acceptance range, with averages of 3.4% and 8.3% by the Q-Plex and 3.5% and 4.1% by the xMAP. The variability of the PanLDH and *Pv*LDH results from DBS samples that were tested by the Q-Plex (Av. 26.3% and Av. 24.6%, respectively) was outside the acceptance range, whereas that from DBS samples evaluated by the xMAP were within the acceptance range (Av. 6.3% and 8.8%, respectively). However, the result of the CV calculated with PanLDH and *Pv*LDH concentrations from the three runs showed an acceptable inter-assay variability for PanLDH (8.3%−14.5%, Av. 9.1%) and a slightly higher variability for *Pv*LDH (5.8%–36.1%, Av. 19.0%). These results suggest overall high reproducible procedures of two platforms over time.

**Table 2:** Inter-assay variability for malaria antigen signal intensity determined using Q-Plex and xMAP platforms

Sample type	Analyte	International standard for *Plasmodium* antigen, CV (%)									
		Q-Plex					xMAP				
		50 IU/mL	25 IU/mL	12.5 IU/mL	6.3 IU/mL	Average	50 IU/mL	25 IU/mL	12.5 IU/mL	6.3 IU/mL	Average
Whole blood	HRP2	1.2	4.8	3.7	9.6	4.8	2.9	4.9	1.5	2.3	2.9
	PanLDH	6.0	3.8	6.3	7.8	6.0	3.9	1.1	10.9	2.7	4.6
	*Pf*LDH	3.3	2.9	0.3	8.1	3.7	8.9	10.0	10.2	5.4	8.6
	*Pv*LDH	0.9	2.1	2.0	2.2	1.7	8.4	10.2	12.9	9.7	9.4
DBS	HRP2	4.9	3.1	2.6	2.9	3.4	6.6	2.5	1.9	2.9	3.5
	PanLDH	34.3	32.6	24.9	13.6	26.3	7.0	4.7	4.5	9.0	6.3
	*Pf*LDH	8.2	10.1	7.3	7.5	8.3	10.8	2.7	1.9	0.8	4.1
	*Pv*LDH	25.6	10.0	16.3	38.4	24.6	12.1	7.7	8.0	6.7	8.8

Samples were tested on multiple days by one operator. Assays for HRP2, PanLDH, and *Pf*LDH were assessed using the WHO international standard *P. falciparum* antigen, while the *Pv*LDH assay was assessed using the WHO international standard *P. vivax* antigen

International standard samples (*n* = 3) at different concentrations

*CV* coefficient of variation, *IU* international unit

### Identification of *P. falciparum* mutants with *hrp2/hrp3* deletions and differentiation of *Plasmodium* species

To determine the effectiveness of the assay system for detecting *P. falciparum mutants* which fail to produce HRP2/HRP3, samples prepared with laboratory *P. falciparum* strains W2 (*hrp2*^+^*hrp3*^+^), Dd2 (*hrp2*^*–*^*hrp3*^+^), and 3BD5 (*hrp2*^*–*^*hrp3*^*–*^) were tested for the presence or absence of HRP2 and *Pf*LDH using the established cutoff values. Both the Q-Plex and the xMAP identified *P. falciparum* parasites with *hrp2* or *hrp2/hrp3* deletions in whole blood with 100% positive agreement through evaluating the reactivity patterns for both HRP2 and HRP3, as well as *Pf*LDH (Table 3). However, the Q-Plex appeared to be less effective than the xMAP for identification of parasites with an *hrp2* deletion (80% for Q-Plex and 100% for the xMAP in detecting Dd2) and with *hrp2/hrp3* deletions (40% for Q-Plex and 80% for the xMAP in detecting 3BD5) in DBS.

**Table 3:** Identification of *P. falciparum* mutants with *hrp2/hrp3* deletions

Sample type	*P. falciparum* strain	Number of samples	% Positive agreement			
			Q-Plex		xMAP	
			*Wild type*	*hrp2/hrp3* deletions	*Wild type*	*hrp2/hrp3* deletions
Whole blood	W2 (*hrp2*^+^*hrp3*^+^)	5	100	NA	100	NA
	Dd2 (*hrp2*^*–*^*hrp3*^+^)	5	100	NA	100	NA
	3BD5 (*hrp2*^*–*^*hrp3*^*–*^)	5	100	100	100	100
DBS	W2 (*hrp2*^+^*hrp3*^+^)	5	100	NA	100	NA
	Dd2 (*hrp2*^*–*^*hrp3*^+^)	5	80	NA	100	NA
	3BD5 (*hrp2*^*–*^*hrp3*^*–*^)	5	40	40	80	80

The established cutoff values were used to determine whether a sample tests positive and negative for HRP2 and *Pf*LDH antigens. *P. falciparum* infection was determined by the test results with the presence of HRP2 or/and *Pf*LDH above the cutoff. The *hrp2/hrp3* deletions were identified by the test results that showed no detectable HRP2 in the presence of *Pf*LDH above the cutoff. The cutoff was determined by the concentration yielding 99.5% or more diagnostic specificity for the Q-Plex and the mean of negative controls + 3 standard deviation for the xMAP

*NA* not applicable

**Fig. 2:**
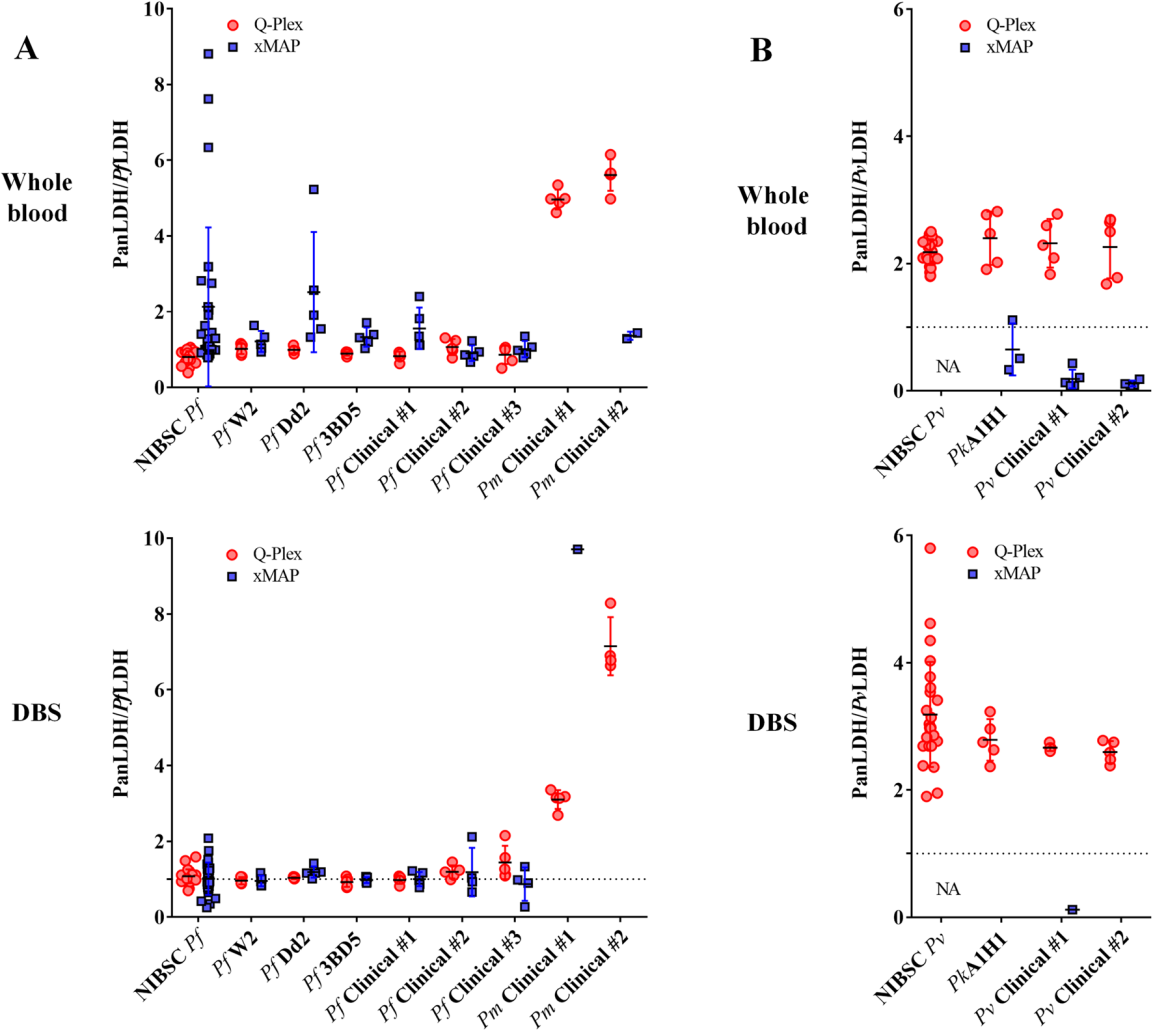
Reactivity to pLDH proteins. Depicted are dot plots for ratios of PanLDH/*Pf*LDH or PanLDH/*Pv*LDH for samples of each subset as indicated. PanLDH standards used in the Q-Plex and xMAP were calibrated against the assay specific *Pf*LDH standards. The samples showing antigen-negative results were excluded from this analysis. *NA* not available

Both the Q-Plex and xMAP platforms detect *P. falciparum* in whole blood with high positive agreement (100%), with the xMAP having a higher sensitivity than the Q-Plex for *P. vivax* infection (Table 4). The Q-Plex shows relatively lower percent positive agreement compared to the xMAP on DBS for *P. falciparum* and *P. vivax* (Table 4). Interestingly, both the Q-Plex and xMAP misidentified *P. malariae* and *P. knowlesi* as *P. falciparum* and *P. vivax* due to cross-reactivity of pLDH from these species against *Pf*LDH- and *Pv*LDH-specific assays, respectively (Table 4). Notably, pLDH from these species showed different reactivity against these antibodies depending on the platforms, as demonstrated by the ratio of concentration between PanLDH to the species-specific pLDH (PanLDH/*Pf*LDH or PanLDH/*Pv*LDH) (Fig. 2).

**Table 4:** Classification of *Plasmodium* species

Sample type	Species	Number of samples	Q-Plex		xMAP	
			% Positive agreement	Classification (number of positive)	% Positive agreement	Classification (number of positive)
Whole blood	*P. falciparum*	15	100	*Pf (15)*	100	*Pf (15)*
	*P. vivax*	10	80	*Pv (8)*	100	*Pv (10)*
	*P. malariae*	10	0	*Pf (3)*	0	*–*
	*P. knowlesi*	5	0	*Pv (3)*	0	*Pv (3)*
DBS	*P. falciparum*	15	33.3	*Pf* (5)	100	*Pf (15)*
	*P. vivax*	10	30	*Pv (3)*	80	*Pv (8)*
	*P. malariae*	10	0	*Pf (2)*	0	–
	*P. knowlesi*	5	0	–	0	*–*

The established cutoff values were used to determine positive and negative values for HRP2, *Pf*LDH, and *Pv*LDH antigens. *P. falciparum* and *P. vivax* infections were identified by test results with concentrations of HRP2 or *Pf*LDH, and *Pv*LDH that were above the cutoff

### Agreement between two multiplex platforms

**Fig. 3:**
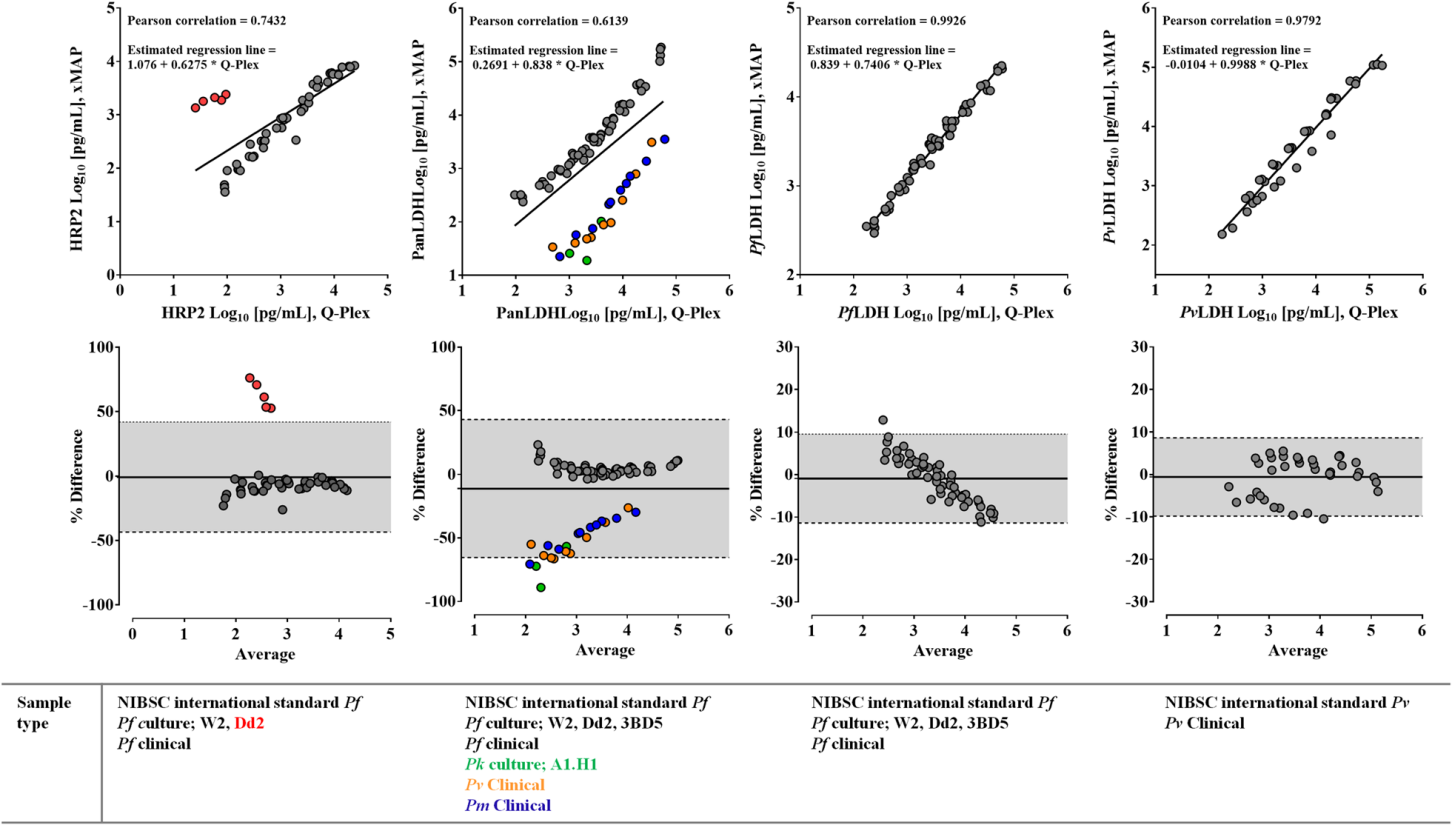
Comparative analyses of HRP2, PanLDH, *Pf*LDH, and *Pv*LDH in whole blood samples. **A** Linear regression scatter plots were generated from log transformed data for the WHO international standard *P. falciparum* and *P. vivax* antigens, *P. falciparum* culture, and clinical samples in whole blood. The equation of regression line and Pearson correlation *r* value were analysed using the paired sets of data for each analyte. **B.** Bland–Altman plots comparing the Q-Plex and xMAP assays for the aforementioned antigens. The mean difference between antigens’ concentrations (solid line) measured by the Q-Plex and xMAP and the 95% confidence interval of the difference (dotted lines) are shown. The samples that are far from a regression line are indicated as circles in plots and names of sample types in colors. Data from 3BD5 samples were excluded, as HRP2 was absent in these samples

**Fig. 4:**
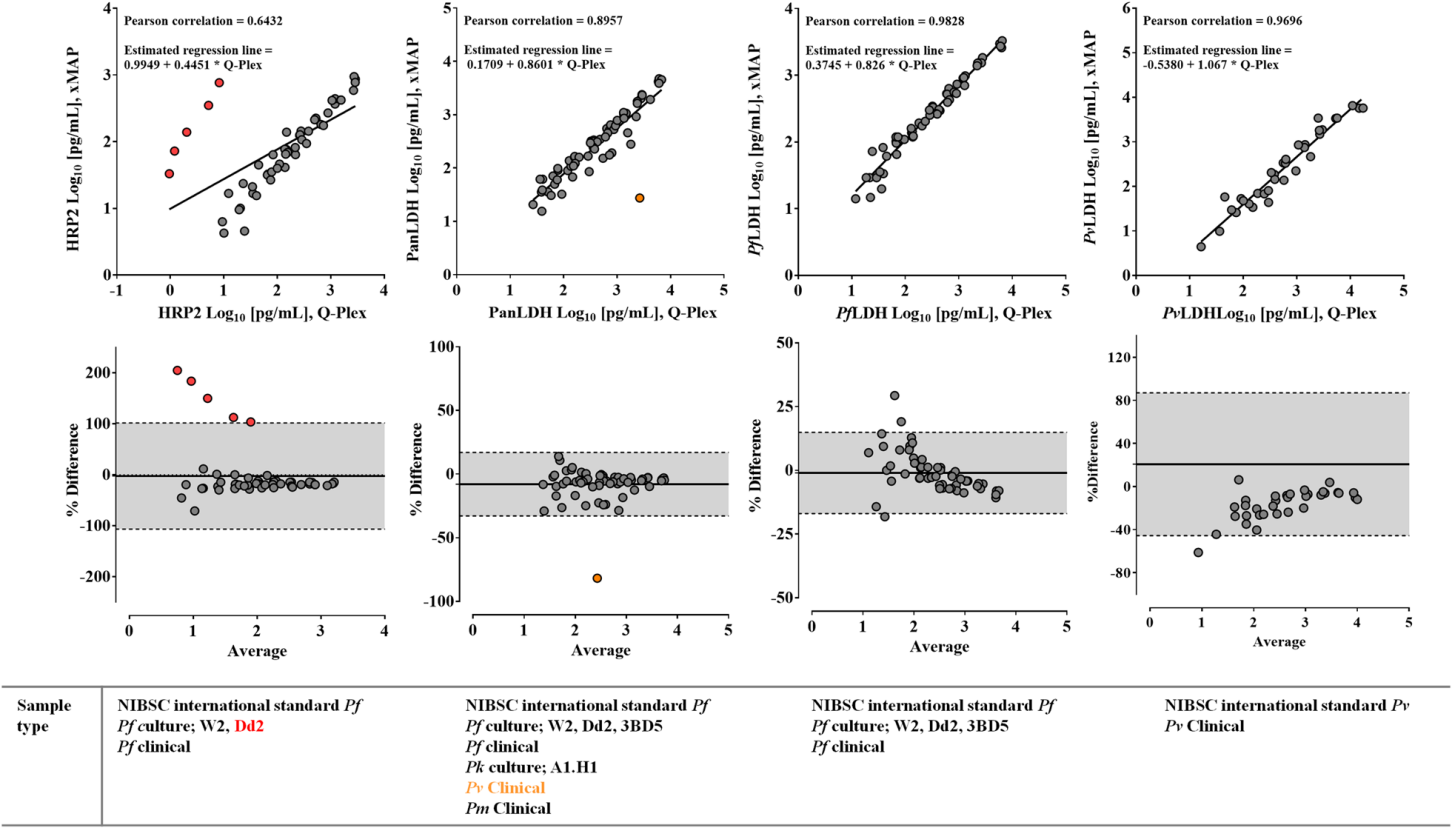
Comparative analyses of HRP2, PanLDH, *Pf*LDH, and *Pv*LDH in DBS samples. **A** Linear regression scatter plots were generated from log transformed data for the WHO international standard *P. falciparum* and *P. vivax* antigens, *P. falciparum* culture, and clinical samples in DBS. The equation of regression line and Pearson correlation were analysed using the paired sets of data for each analyte. **B** Bland–Altman plots comparing the Q-Plex and xMAP assays for the aforementioned antigens. The mean difference between antigens’ concentrations (solid line) measured by the Q-Plex and xMAP and the 95% confidence interval of the difference (dotted lines) are shown. The samples in a cluster off a regression line are indicated as circles in plots and names of sample types in colors. Data from 3BD5 samples were excluded as HRP2 was absent in these samples

The Pearson correlation coefficient to determine the agreement between the Q-Plex and xMAP for HRP2 showed moderate and poor positive correlations between HRP2 antigen levels in whole blood (*r* = 0.7432; Fig. 3) and DBS (*r* = 0.6432; Fig. 4), respectively. There was a notable discrepancy of the HRP2 results between the Q-Plex and xMAP assays with the dilution series of the *hrp2*^*–*^*hrp3*^+^ laboratory strain Dd2. In these samples, the concentration of HRP3, which may cross-react with HRP2 detector antibodies, gave a higher signal with HRP2-specific assay of the xMAP compared to the Q-Plex, which showed minimal cross-reactivity with HRP3. This is also shown in assay-specific HRP2 concentration against parasite density plots (Additional file 1: Figure S1). A systematic trend of higher assay values for *Pf*LDH and *Pv*LDH was observed with the Q-Plex testing in both whole blood and DBS. However, a strong agreement between the Q-Plex and xMAP was observed with *Pf*LDH antigen, as demonstrated by Pearson correlation coefficient *r* values that were 0.9926 and 0.9792 for *Pf*LDH and *Pv*LDH in whole blood and 0.9792 and 0.9696 in DBS. As for PanLDH assay, the Pearson *r* value was the lowest at 0.6139 in whole blood, having two obvious clusters in a plot, but it improved to 0.8957 in DBS. The Bland–Altman plots revealed a systematic trend of difference between the Q-Plex and xMAP assays for PanLDH that was derived from the specific subsets of samples, such as *P. knowlesi* culture and clinical *P. vivax* and *P. malariae,* suggesting that two platforms may have different binding reactivities against pLDH proteins from these parasites (Fig. 3).

### Normalization of the quantitative results against the WHO international standards

**Fig. 5:**
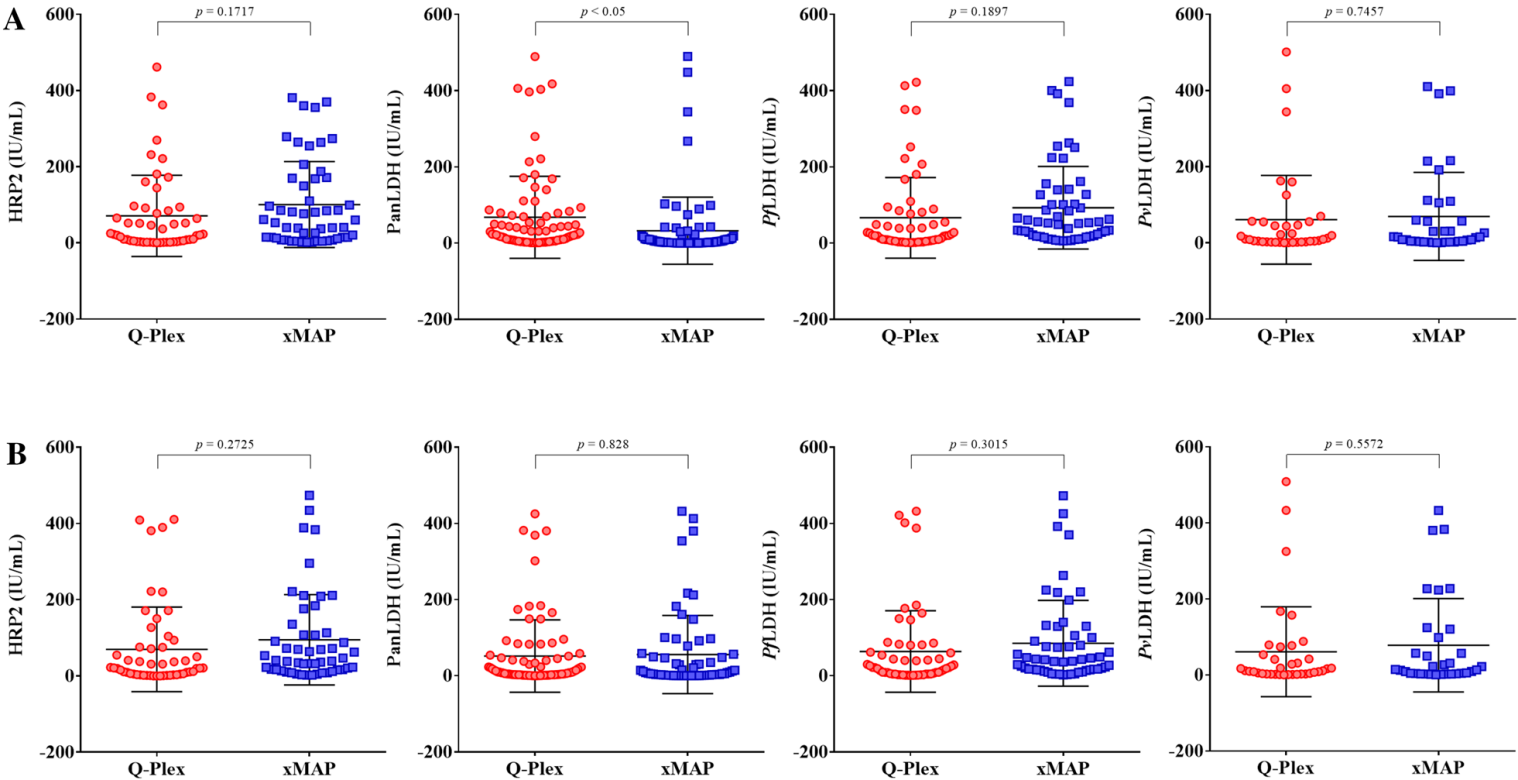
Comparison of *Plasmodium* antigen levels adjusted in international units. Dot plots depict the distribution of standardized concentration measures in samples of (**A**) whole blood and (**B**) DBS. The t-test was performed for statistical analysis. Statistically significant at *p* < 0.05

In this study, the concentration of antigen measured by two immunoassay methods was plotted against the WHO international standard antigens (Fig. 1), and the slope of each linear regression line was obtained for the “conversion factors” (Additional file 1: Table S3). Subsequently, these conversion factors were used to re-analyse the original concentration data, reporting the amount of *Plasmodium* antigens in IU/mL. When the normalized antigen levels in the IU/mL were compared between the Q-Plex and xMAP, there were similar distribution profiles between the two for HRP2, *Pf*LDH, and *Pv*LDH in both whole blood and DBS (Fig. 5). However, a statistically significant difference between the two was found for PanLDH in whole blood (*p* < 0.05) but not for that in DBS (*p* = 0.828). Similarly, correlation analysis using normalized antigen concentration values showed a strong correlation between the Q-Plex and xMAP for *Pf*LDH and *Pv*LDH antigens but poor to moderate correlation between the two for HRP2 or PanLDH in whole blood and DBS, demonstrating some variation in binding reactivities against HRP2/HRP3 or PanLDH (Additional file 1: Figure S2).

## Discussion

Several malaria multiplex platforms have been developed to target key biomarkers, including HRP2, *Pf*LDH, *Pv*LDH, PanLDH, and pAldo [8, 9, 13, 22, 23]. These have proven valuable in clinical studies for understanding the biology of parasites, evaluating the performance of RDTs, and documenting the decay rates of *Plasmodium* antigens, among other uses. The ability to compare data across the different assays would greatly enhance the value of the results generated with these multiplexed assays. The present study sought to assess how two of these multiplex immunoassays compare in terms of the quantitative results of those *Plasmodium* antigens, their agreement in identifying *Plasmodium* species, and whether the quantitative results can be compared across platforms.

In this study, the assay performance of two malaria multiplex platforms, the Q-Plex and xMAP, which allow the quantification of four common malaria antigens—HRP2, *Pf*LDH, *Pv*LDH, and PanLDH—in biological samples, were compared. The xMAP assay is an in-house research assay, while the Q-Plex assay is a commercial off-the-shelf product. Both platforms are considered moderate throughput screening assays, with similar procedures and assay time to results. The xMAP provides a flexible and affordable platform by which to measure parasite antigen concentrations from a per specimen perspective once the instrumentation has been purchased, but it requires laboratories to individually source their assays reagents and validate them. The Q-Plex platform provides a commercial kit for which all components are already included and validated, but the per specimen cost is higher and there is no ability to rapidly add new assays to the platform. There are additional differences in physical characteristics, including assay format (plate-based versus bead-based), assay volume (50 µL versus 100 µL), required sample volume (12.5 µL versus 50 µL for the single target assay), and detection and reporting methods (chemiluminescence versus fluorescence, pixel intensity versus net median fluorescent intensity), which are summarized in Additional file 1: Table S1.

To assess the compatibility of data generated across both platforms, paired sample panels in whole blood and DBS were created to conduct an objective analysis of the performance of both platforms. The identical sample panels were independently analysed by two laboratories using the Q-Plex and xMAP assays. The inclusion of the WHO international standards for *P. falciparum* and *P. vivax* antigens allows standardization of the outputs of the two platforms in international units. Additionally, inclusion of *hrp2/hrp3* deleted parasite strains and different *Plasmodium* species screened in these assay platforms allowed for a better understanding of differences in overall performance as well as the quantitative measurements derived from the two platforms. Given the differences in the assay dynamic ranges for the respective antigens between the two platforms, the sample testing was performed as blind experiments at two dilutions—neat and a second dilution. Excellent linearity was observed when using the WHO international standards in a range of 1.6–400 IU/mL and assay-specific calibrators for both the Q-Plex and xMAP assays, with R^2^ values > 0.9 for all antigen-specific assays. However, as demonstrated by different slope values in the linear regression fits for each analyte, the two platforms do behave differently. This is most likely because different standard materials are used to calibrate the antigen concentrations in the two platforms. Overall, the inter-assay precision expressed as CV (calculated using the signal value; acceptable ≤ 15%) were acceptable across antigens and platforms. For the Q-Plex in DBS, the PanLDH and *Pv*LDH CV values exceeded the acceptable range and only the PanLDH signal when analysed in concentration units fell within the acceptable range. These data are indicative of a good level of reproducibility of these assays and met the predetermined assay acceptance criteria, with the Q-Plex not performing as well on DBS.

Both assays effectively identified laboratory *P. falciparum* parasite (3BD5) with deletion of *hrp2* and *hrp3* genes in addition to classifying *Plasmodium* species in clinical specimens using three detection reagents targeting HRP2, *Pf*LDH, and *Pv*LDH. In particular, the xMAP showed a higher percent positive agreement for identification of different *Plasmodium* infections, when the established cutoff value for each of these biomarkers was used.

*Pf*LDH and *Pv*LDH concentrations measured by the Q-Plex showed a strong positive correlation with those measured by the xMAP. However, HRP2 or PanLDH concentration data from the two showed a moderate to poor correlation. Overall, the Q-Plex showed 2.4-fold higher HRP2 concentration estimation compared to the xMAP. This cannot be attributed to HRP3, since a significant difference in the HRP2 assay between the two platforms is the relative response to the *P. falciparum* strain Dd2, which still expresses HRP3 but has an *hrp2* deletion. Specifically, the Q-Plex HRP2 assay has minimal cross-reactivity with HRP3, but the xMAP assay does react strongly with HRP3. These differences are relevant to take into account when these assays are used to support RDT evaluations with clinical samples with *hrp2/hrp3* deletions. Different reactivities to HRP3 have also been observed in commercially available RDTs [24, 25].

Antibodies for pLDH have very different specificity profiles such that RDTs and ELISA assays developed for *Pf*LDH and *Pv*LDH behave very differently across the other species of *Plasmodium* [26–30]. On the Q-Plex platform, the pLDH proteins from *P. malariae* and *P. knowlesi* were found to cross-react to *Pf*LDH and *Pv*LDH assays, respectively, whereas on the xMAP assay only *P. knowlesi* was shown to cross-react with the *Pv*LDH assay. These differences in cross-reactivity also extend to the PanLDH signal. Substantial differences were observed between the Q-Plex and the xMAP, in the PanLDH signal as well as species-specific pLDH signal as shown by the ratio of both measurements. The PanLDH signal in the Q-Plex is calibrated against the *Pf*LDH standard, and thus the PanLDH/*Pf*LDH ratio is inherently 1. This study found the ratio of PanLDH/*Pf*LDH to be close to 1 across all *P. falciparum* samples including the WHO international standard for *P. falciparum* antigen (0.805 ± 0.158), *P. falciparum* strains (0.892 ± 0.052–1.02 ± 0.134), and clinical samples (0.828 ± 0.119–1.068 ± 0.212). In contrast, the Q-Plex shows much higher PanLDH/*Pf*LDH ratios with two *P. malariae*–infected samples (4.965 ± 0.262 and 5.61 ± 0.416), which allows the Q-Plex to differentiate these from *P. falciparum* infections. The PanLDH/*Pv*LDH ratios in *P. knowlesi* samples (2.398 ± 0.419) tested by using the Q-Plex were similar to those from two *P. vivax* subsets of samples (2.318 ± 0.382, 2.26 ± 0.49). The binding patterns appear to be similar to those previously reported with the Q-Plex [31, 32]. Thus in *P. vivax* and *P. knowlesi* co-endemic populations, the Q-Plex would not be able to differentiate between the two infections without further development. Similar consistent PanLDH/*Pf*LDH and PanLDH/*Pv*LDH ratios for *P. knowlesi* and *P. malariae* were not observed with the xMAP, and the cross-reactivity requires further investigation. However, the xMAP can differentiate *P. knowlesi* from *P. vivax* infections due to the reduced cross-reactivity in the *P. vivax* assay for *P. knowlesi*.

The PanLDH signal also showed significant variation between the two platforms with the non–*P. falciparum* samples in whole blood behaving significantly differently. Overall, there was a threefold difference in PanLDH concentration as determined by the Q-Plex compared to the xMAP. These variations in PanLDH quantification most likely arise from differences in the choice of detection antibodies resulting in recognition of possibly different target epitopes and calibration standards used for the assays on each platform. These differences will impact the relative PanLDH to species-specific LDH assay signal as well as the absolute quantification of PanLDH across both assays.

The study also explored normalization of the assay signals with the WHO international standards for *P. falciparum* and *P. vivax* antigen and expression of the concentration in IU/mL. The antigen distribution plots between the two assays in quantification of HRP2, *Pf*LDH, and *Pv*LDH show that normalized data can be comparable between assays when the results are expressed in IU/mL. Further work is required to understand the feasibility of using international standards to normalize the PanLDH assay.

## Conclusion

The data indicate that both the Q-Plex and xMAP showed good performance in detecting wild type *P. falciparum*, *hrp2/hrp3*-deleted *P. falciparum* mutants, and other *Plasmodium* species, but key differences were also observed. The study also shows agreement in quantification of HRP2, *Pf*LDH, and *Pv*LDH data obtained by the Q-Plex and xMAP platforms once normalization into international units with the WHO international standards for *P. falciparum* and *P. vivax* antigens is conducted. This significant finding enables comparison and utilization of results across malaria antigen quantification platforms.

## Supplementary Information

**Additional file 1:** **Table S1.** Comparison of two multiplex platforms for the quantitative assessment of malaria antigens. **Table S2.** Characteristics of the Q-Plex and xMAP assays. **Table S3.** Linear relation between the WHO international standard *P. falciparum* and *P. vivax* antigens and assay concentration results in whole blood or DBS. Regression analysis showed the fit of the data with R^2^ values that met the acceptance criteria (R^2^ ≥ 0.85). One operator conducted three experiments over a week. **Figure S1.** HRP2 concentration in (**A**) whole blood and (**B**) DBS plotted against the parasitemia. Three subsets of dilution-series samples composed of different *P. falciparum* laboratory strains (W2, Dd2, and 3BD5) were tested by using the Q-Plex and xMAP. Measured concentration was plotted on the y-axis against parasitemia (par/mL) on the x-axis. Red and blue symbols and regression lines represent samples spiked with different strains and test types. **Figure S2.** Correlation analysis. Correlation plots of antigen concentration corrected against the WHO international standard *P. falciparum *and *P. vivax *antigens, *P. falciparum* culture, and clinical samples in (**A**) whole blood and (**B**) DBS. The Pearson correlation *r* value and the equation of the regression line obtained from analysis using log transformed data are shown. Outlier sample sets were color-coded.

## Data Availability

Available from the corresponding author on request.

## Abbreviations

Av.: Average
CRP: C-reactive protein
CV: Coefficient of variation
DBS: Dried blood spot
ELISA: Enzyme-linked immunosorbent assay
HRP2: Histidine-rich protein 2
HRP3: Histidine-rich protein 3
LB: Luminex buffer
LDH: Lactate dehydrogenase
LLOD: Lower limit of detection
LLOQ: Lower limit of quantification
MFI: Median fluorescence intensity
NIBSC: National institute for biological standards and control
pAldo: *Plasmodium* aldolase
PanLDH: Pan-specific pLDH
PBS: Phosphate-buffered saline
*Pf*LDH: *P. falciparum*–specific pLDH
pLDH: *Plasmodium* lactate dehydrogenase
*Pv*LDH: *P. vivax*–specific pLDH
R^2^: R squared, coefficient of determination
RDT: Rapid diagnostic test
rpm: Revolutions per minute
SD: Standard deviation
ULOQ: Upper limit of quantification
WB: Whole blood
WHO: World Health Organization
