# Supplementary material for: Comparison of two malaria multiplex immunoassays that enable quantification of malaria antigens
Source: Malar J. 2022 Jun 7;21:176. doi: 10.1186/s12936-022-04203-9 (PMC9171962; doi:10.1186/s12936-022-04203-9)
Supplement: Supplementary file 1 — Additional file 1: Table S1. Comparison of two multiplex platforms for the quantitative assessment of malaria antigens. Table S2. Characteristics of the Q-Plex and xMAP assays. Table S3. Linear relation between the WHO international standard P. falciparum and P. vivax antigens and assay concentration results in whole blood or DBS. Regression analysis showed the fit of the data with R2 values that met the acceptance criteria (R2 ≥ 0.85). One operator conducted three experiments over a week. Figure S1. HRP2 concentration in (A) whole blood and (B) DBS plotted against the parasitemia. Three subsets of dilution-series samples composed of different P. falciparum laboratory strains (W2, Dd2, and 3BD5) were tested by using the Q-Plex and xMAP. Measured concentration was plotted on the y-axis against parasitemia (par/mL) on the x-axis. Red and blue symbols and regression lines represent samples spiked with different strains and test types. Figure S2. Correlation analysis. Correlation plots of antigen concentration corrected against the WHO international standard P. falciparum and P. vivax antigens, P. falciparum culture, and clinical samples in (A) whole blood and (B) DBS. The Pearson correlation r value and the equation of the regression line obtained from analysis using log transformed data are shown. Outlier sample sets were color-coded. [file 12936_2022_4203_MOESM1_ESM.docx]

**Comparison of two malaria multiplex immunoassays that enable quantification of malaria antigens**

**Authors: Ihn Kyung Jang ^1#^, Alfons Jimenez^2,3^, Andrew Rashid^1^, Rebecca Barney^1^, Allison Golden^1^, Xavier C. Ding^4^, Gonzalo J. Domingo^1*^, Alfredo Mayor^2,3,5,6*^**

^1^ Diagnostic group, PATH, 2201 Westlake Ave, Suite 200, Seattle, WA, 98121, USA

^2^ ISGlobal, Barcelona Institute for Global Health, Hospital Clínic - Universitat de Barcelona, Carrer Rosselló 153 (CEK Building), E-08036 Barcelona, Spain

^3^ Spanish Consortium for Research in Epidemiology and Public Health (CIBERESP), Av. Monforte de Lemos, 3-5, Pabellón 11, 28029 Madrid, Spain

**^4^** The Foundation for Innovative New Diagnostics, Campus Biotech, 9 Chemin des Mines - 1202 Geneva, Switzerland

^5^ Centro de Investigação em Saúde de Manhiça Rua 12, Cambeve CP 1929, Maputo, Mozambique

^6^ Department of Physiologic Sciences, Faculty of Medicine, Universidade Eduardo Mondlane, Maputo, Mozambique

^*^ Both authors contributed equally.

^#^ Correspondence author: Address: PATH, 2201 Westlake Ave, Suite 200, Seattle, WA, 98121, USA. E-mail: [ikjang@path.org](mailto:ikjang@path.org). Phone: 206-302-4738

**Additional file 1: Table S1.** Comparison of two multiplex platforms for the quantitative assessment of malaria antigens.

| **Parameter** | **Q-Plex platform** | **xMAP platform** |
| --- | --- | --- |
| Assay format | Plate-based | Bead-based |
| Number of analytes | 5 analytes/well (HRP2, *Pf*LDH, *Pv*LDH, PanLDH, CRP) | Two assays: 4 analytes/well (HRP2, *Pf*LDH, PanLDH, pAldo; 1 analyte/well (*Pv*LDH) |
| Assay volume | 50 μL | 100 μL |
| Sample volume | 12.5 μL for 5 analytes tested in neat; diluted with assay diluent by 4-fold | 50 μL for 5 analytes tested in neat; diluted with assay diluent by 5-fold |
| Detection device | Q-view ^TM^ Imager; benchtop | Luminex 200^TM^ or FLEXMAP® analyzer; benchtop |
| Throughput | Moderate | Moderate |
| Operator skill | Basic | Moderate |
| Training time | A full day | A full day |
| Hand-on time | 0.5 hour/plate | 0.5 hour/plate |
| Assay length | 3.5 hours | 3.0 hours |
| Detection | Chemiluminescence | Fluorescence |
| Result reported | Pixel intensity and concentration | Net MFI (median fluorescent intensity) and concentration |
| Chemistry | Biotinylated detection malaria specific antibodies | Biotinylated detection malaria specific antibodies |
| Sensitivity | High^a^ | High |
| Specificity | High^a^ | High |

^a^ Validated using PCR-confirmed clinical samples from asymptomatic and symptomatic individuals.

**Additional file 1: Table S2.** Characteristics of the Q-Plex and xMAP assays.

| **Platform** | **Analyte** | **Cutoff (pg/mL)** | **LLOD** **(pg/mL)** | **LLOQ** **(pg/mL)** | **ULOQ** **(pg/mL)** |
| --- | --- | --- | --- | --- | --- |
| **Q-Plex** | **HRP2** | 6.9 | 0.6 | 0.7 | 49,300 |
|  | **PanLDH** | 49.0 | 16.6 | 18.1 | 1,350,840 |
|  | ***Pf*LDH** | 553.6 | 4.3 | 6.9 | 483,420 |
|  | ***Pv*LDH** | 314.8 | 4.0 | 5.3 | 364,400 |
| **xMAP** | **HRP2** | 26.6 | 6.9 | 14.8 | 79,480 |
|  | **PanLDH** | 90.5 | 45.7 | 90.5 | 646,980 |
|  | ***Pf*LDH** | 110.3 | 46.6 | 94.3 | 441,900 |
|  | ***Pv*LDH** | 25.0 | 20.6 | 40.8 | 170,380 |

Abbreviations: LLOD, lower limit of detection; LLOQ, lower limit of quantification; ULOQ, upper limit of quantification.

**Additional file 1: Table S3.** Linear relation between the WHO international standard *P. falciparum* and *P. vivax* antigens and assay concentration results in whole blood or DBS. Regression analysis showed the fit of the data with R^2^ values that met the acceptance criteria (R^2^ ≥ 0.85). One operator conducted three experiments over a week.

|  | | **Q-Plex** | | | **xMAP** | | |
| --- | --- | --- | --- | --- | --- | --- | --- |
|  |  | **Trendline** | **R^2^** | **International standard calibration (equivalent to 1 IU); pg** | **Trendline** | **R^2^** | **International standard calibration (equivalent to 1 IU); pg** |
| **Whole blood** | **HRP2** | *y=51.79x–168.3* | 0.9797 | 52 ^a^ | *y=21.55x+443.3* | 0.9360 | 22 |
|  | **PanLDH** | *y=124.5x–897.8* | 0.9875 | 125 ^a^ | *y=382.0x–8141* | 0.9037 | 382 |
|  | ***Pf*LDH** | *y=141.6x–545.2* | 0.9885 | 142 ^a^ | *y=53.07x+1278* | 0.9778 | 53 |
|  | ***Pv*LDH** | *y=343.2x–3334* | 0.9461 | 343 ^b^ | *y=0.2736x+1.176* | 0.9980 | 0.3 ^c^ |
| **DBS** | **HRP2** | *y=6.890x–50.41* | 0.9886 | 7 | *y=2.123x+7.329* | 0.9890 | 2 |
|  | **PanLDH** | *y=15.57x –62.36* | 0.9938 | 16 | *y=11.22x–8.570* | 0.9947 | 11 |
|  | ***Pf*LDH** | *y=15.26x–98.95* | 0.9910 | 15 | *y=7.335x+102.6* | 0.9796 | 7 |
|  | ***Pv*LDH** | *y=34.10x–326.5* | 0.9215 | 34 | *y=15.27x+48.61* | 0.9899 | 15 |

^a^ Calibrated by WHO international standard *P. falciparum* antigen.

^b^ Calibrated by WHO international standard *P. vivax* antigen.

**Additional file 1: Figure S1.** HRP2 concentration in (**A**) whole blood and (**B**) DBS plotted against the parasitemia. Three subsets of dilution-series samples composed of different *P. falciparum* laboratory strains (W2, Dd2, and 3BD5) were tested by using the Q-Plex and xMAP. Measured concentration was plotted on the y-axis against parasitemia (par/mL) on the x-axis. Red and blue symbols and regression lines represent samples spiked with different strains and test types.

**
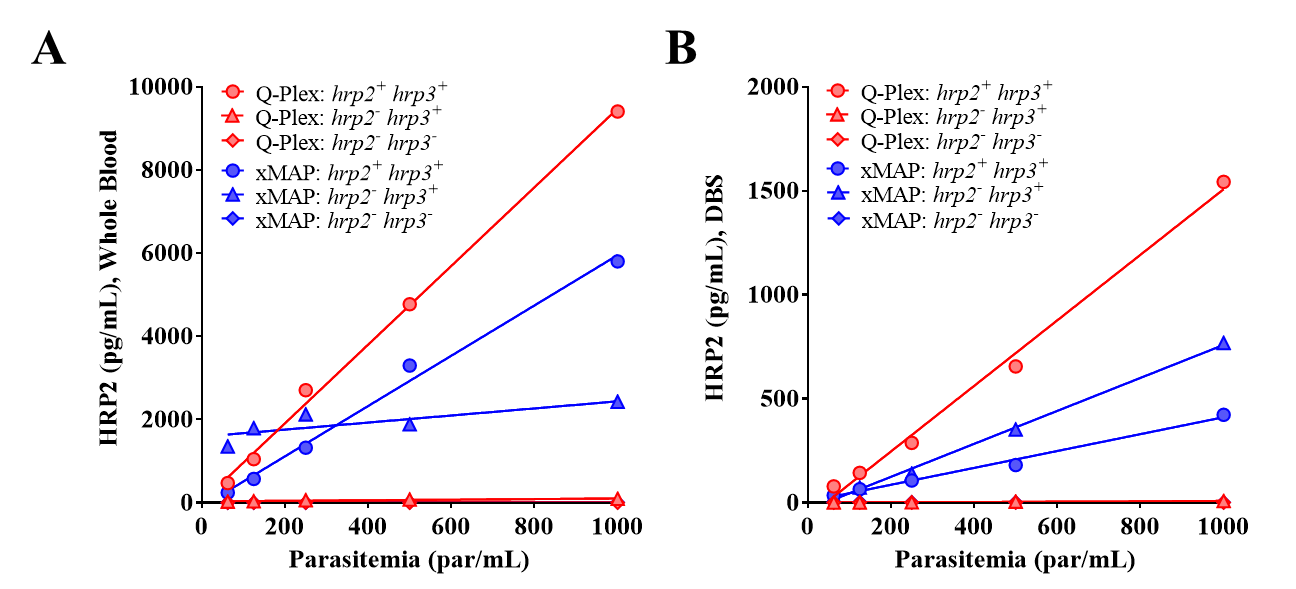
**

**Additional file 1: Figure S2 Correlation analysis.** Correlation plots of antigen concentration corrected against the WHO international standard *P. falciparum* and *P. vivax* antigens, *P. falciparum* culture, and clinical samples in (**A**) whole blood and (**B**) DBS. The Pearson correlation *r* value and the equation of the regression line obtained from analysis using log transformed data are shown. Outlier sample sets were color-coded.

**
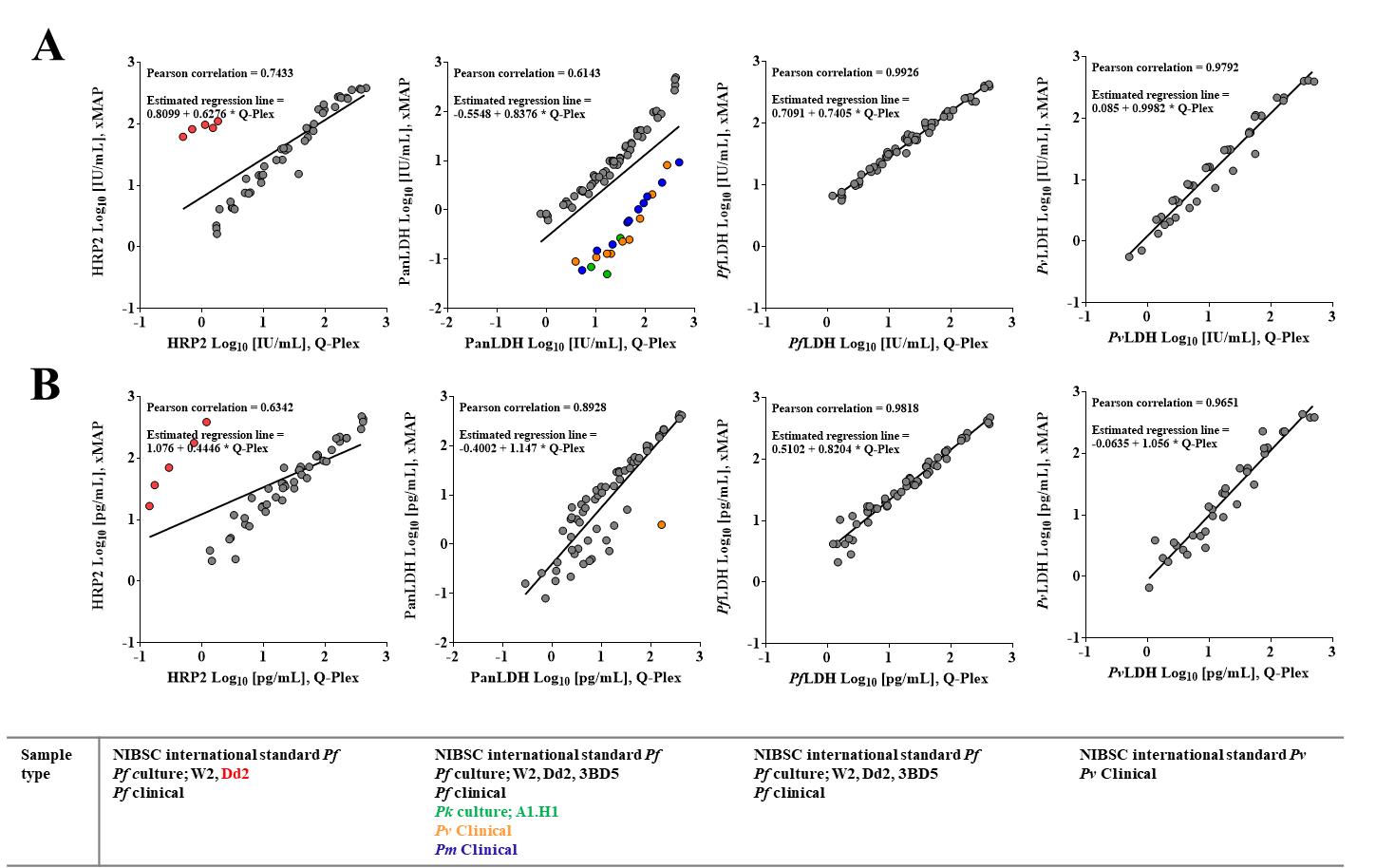
**
